# The use of implementation science theories, models, and frameworks in implementation research for medicinal products: A scoping review

**DOI:** 10.1186/s12961-024-01102-0

**Published:** 2024-01-29

**Authors:** Meredith Y. Smith, Bridget Gaglio, Milena Anatchkova

**Affiliations:** 1 Evidera, Inc., Bethesda, MD United States of America; 2 Department of Regulatory and Quality Sciences, School of Pharmacy, University of Southern California, Los Angeles, CA United States of America

**Keywords:** Implementation science, Dissemination, Knowledge translation, Pharmaceuticals, Drug development, Theories, Models, Frameworks, Implementation strategies

## Abstract

**Background:**

The uptake, adoption and integration of new medicines and treatment regimens within healthcare delivery can take a decade or more. Increasingly, implementation science (IS) research is being used to bridge this gap between the availability of new therapeutic evidence and its actual application in clinical practice. Little is known, however, about the quality of IS research in this area, including the degree to which theories, models and frameworks (TMFs) are being used. The objective of this study was to conduct a scoping review of the use of TMFs in implementation research involving medicinal products.

**Methods:**

A search was conducted for English language abstracts and manuscripts describing the application of TMFs in IS studies for medicinal products. Eligible publications were those published between 1 January 1974 and 12 December 2022. All records were screened at the title and abstract stage; included full-text papers were abstracted using data extraction tables designed for the study. Study quality was appraised using the Implementation Research Development Tool.

**Results:**

The initial scoping search identified 2697 publications, of which 9 were ultimately eligible for inclusion in the review. Most studies were published after 2020 and varied in their objectives, design and therapeutic area. Most studies had sample sizes of fewer than 50 participants, and all focused on the post-marketing phase of drug development. The TMF most frequently used was the Consolidated Framework for Implementation Research (CFIR). Although most studies applied all TMF domains, TMF use was limited to instrument development and/or qualitative analysis. Quality appraisals indicated the need for engaging patients and other stakeholders in the implementation research, reporting on the cost of implementation strategies, and evaluating the unintended consequences of implementation efforts.

**Conclusions:**

We found that few IS studies involving medicinal products reported using TMFs. Those that did encompassed a wide variety of therapeutic indications and medicinal products; all were in the post-marketing phase and involved limited application of the TMFs. Researchers should consider conducting IS in earlier phases of drug development and integrating the TMFs throughout the research process. More consistent and in-depth use of TMFs may help advance research in this area.

**Supplementary Information:**

The online version contains supplementary material available at 10.1186/s12961-024-01102-0.

## Contribution to the Literature

- This scoping review examined the quality of research in an emerging area of implementation science (IS): the uptake, adoption and integration of medicinal products into clinical care.
- Of the studies meeting our review criteria, only a few were applying theories, models or frameworks (TMFs). All of the studies were in the post-marketing period, and TMF application was limited. Patient and other stakeholder engagement was lacking, and neither the costs nor the unintended consequences of implementing specific IS strategies were reported.
- IS research focusing on medicines could benefit from greater and more in-depth use of TMFs to improve the evidence base.

## Introduction

The development of a new pharmaceutical product is a lengthy, complex and highly expensive endeavour. Recent estimates indicate that it can take up to 17 years to bring a new medicine to market at an average cost of approximately $1.1 billion per product [1, 2]. Advanced administration and delivery technologies, a burgeoning aspect of medicinal product innovation, promise to yield more therapeutically effective drug formulations in the future, but with no commensurate improvements in development costs or increased speed of translation to market [1, 3].

Although the attainment of marketing authorization approval is a critical milestone in the drug product life cycle, it is no guarantee of a product’s actual use in clinical practice [4]. In fact, scale-up (that is, the uptake, adoption and integration of new, evidence-based treatments within healthcare systems) can itself take a decade or more [5]. The medical literature is replete with examples of this phenomenon, including the persistently low prescribing of steroids to women in premature labour despite evidence of their beneficial effect on fetal lung surfactant [6], the slow uptake in prescribing of prophylactic anticoagulants for orthopaedic surgery patients [7], and the 13-year delay between the demonstrated effectiveness of thrombolytic treatment for myocardial infarction and its advocacy by key opinion leaders [8].

Sustaining the use of these innovations over time represents yet another challenge, especially in light of known or emerging data regarding a product’s safety profile. Many new therapies carry important risks related to their mechanism of action and/or mode of administration, risks that are required to be minimized via specific drug safety interventions or programs. Such risk minimization programs can entail a wide variety of activities (for example, education and training of healthcare professionals and patients, controlled product distribution) to ensure safe and appropriate use of the medicine and to enhance its benefit–risk profile. Regulators are increasingly demanding evidence to demonstrate that these programs have been implemented with fidelity, are effective in minimizing the targeted risks, are not unduly burdensome on the healthcare system, and are continuing to operate throughout the duration of the post-marketing commitment [9, 10]. Research to date, however, indicates that many such programs are not being implemented fully and are thus falling short in terms of intended impact [11–13].

Delays in the uptake, adoption or sustained use of new medicinal products and risk-minimization programs represent significant “gaps” in healthcare delivery. These gaps can lead to suboptimal health outcomes for both individual patients and the population as a whole [14]. Given this, there is growing recognition of the value of implementation science (IS) research in involving medicinal products and therapeutic regimens [15, 16].

As a field, IS focuses on understanding how new healthcare innovations or interventions can be deployed and maintained effectively in a range of different real-world contexts using a variety of theoretically or empirically supported implementation strategies [17]. In the context of medicinal product-related IS research specifically, innovations can include (1) programs (for example, a program to minimize patients’ risk of developing meningococcal infection while using eculizamab) [18], (2) practices and practice guidelines (for example, differential drug treatment recommendations for patients with diabetes and comorbid conditions) [19], (3) procedures (for example, screening for tuberculosis infection before initiation of anti-tumour necrosis factor therapy) [20], (4) pharmaceutical products [for example, pre-exposure prophylaxis to prevent human immunodeficiency virus (HIV) infection] [21], (5) drug administration or delivery technologies (for example, naloxone nasal spray dispensers to counteract the effect of opioid overdose) [22] and (6) regulatory policies and guidances (for example, limiting pack size for paracetamol/acetaminophen products) [23].

Despite its youth, the field of IS has made significant advances both conceptually and methodologically over the past decade, and consensus has emerged regarding what constitutes good practices in study design and conduct [24–28]. These good practices have been developed in response to limitations seen in IS research to date, including that involving medicinal products. Such limitations include misinterpretation of key IS terms or constructs, failure to distinguish between an intervention and its associated implementation strategy/strategies, lack of information regarding how and why specific implementation strategies were selected and the absence of explicit linkages between implementation outcomes and study aims [24, 25]. Additionally, the application of theories, models and frameworks (TMFs), a defining hallmark of high-quality IS research, has been inconsistent or missing altogether [29–33],

Five main types of TMFs have been identified as applicable for IS research. These include process models, determinant frameworks, classic theories, implementation theories and evaluation frameworks [34]. These TMFs can be used for multiple purposes, either singly or in combination, to frame the research questions, specify hypothesized relationships between constructs, identify key constructs that may serve as barriers or facilitators to implementation, select strategies to address barriers, explain causal relationships between a phenomenon and specific outcomes, inform data collection and analysis, specify and predict implementation outcomes, guide implementation or evaluation planning and clarify terminology [35].

To date, one systematic review has examined the content of existing implementation TMFs for use in healthcare [36]. Several scoping reviews regarding the use of implementation TMFs to support uptake of health innovations in specific populations have also been conducted [32, 37]. Little is known, however, regarding the types of TMFs that are being used in IS research involving medicinal products, how and in what ways they have been applied, and for what purpose. The goal of this study was to address this gap in the evidence base.

## Methods

To address our research question regarding the use of TMFs in IS research involving medicinal products, we specified three objectives:

1. To assess the types of studies that have been conducted which reference the use of TMFs, including study purpose, sample characteristics, target medical condition, drug product(s) and study design, and when they are conducted in the product life cycle.
2. To describe how TMFs were used, including which TMFs were used and the rationale for their selection, what domains and/or constructs were applied and for what purpose, and whether any TMF modifications were made.
3. To evaluate the quality of the studies overall in terms of defined IS research quality criteria.

To accomplish these objectives, we conducted a scoping review of the use of TMFs in implementation research involving medicinal products. We chose a scoping review because this is a new area of IS research; a scoping review permits synthesis of findings across a wide range of study types and designs and provides a broad overview of a topic [38]. The conduct and reporting of the scoping review was guided by the Preferred Reporting Items for Systematic Reviews and Meta-Analyses (PRISMA) Scoping Review Guidelines [38]. Human subjects review was not applicable. A study protocol was developed but was not registered, because registration has not been deemed essential for conducting scoping reviews [38].

### Search strategy and eligibility criteria

The search strategy was limited to English language publications and included strings of terms describing IS (that is, “implementation”, “diffusion of innovation” and “translational research”), frameworks (that is, “models”, “theories” and “domains”) and drug-development life cycle (that is, “pharmaceutical”, “medicine” and “pipeline”). The list of search terms used for each database is included in Additional file 1: Table S1. Data sources included Embase, Medline, Cochrane Central Register of Controlled Trials, and Cochrane Database of Systematic Reviews. The final search inclusion period for the first three of these databases was from 1 January 2000 until 12 December 2022, and for the last database (Cochrane Systematic Reviews), it was 1 January 2005–12 December 2022. The search was executed on 13 December 2022. The titles and abstracts of all identified publications were screened and retained for full-text review if they met the following predetermined eligibility criteria:

1. The title or abstract clearly noted use of an implementation TMF.
2. The paper reported results of applying the TMF at any point in the drug-development life cycle.
3. The publication (manuscript or abstract) was in English.

All identified references were screened by two of three reviewers (MA, BG and MYS), decisions on inclusion were compared and any discrepancies were resolved through team discussion including all three reviewers. For the screening of full-text papers, an additional eligibility criterion was used which stipulated that the study presented results of original empirical research only.

### Data abstraction

All articles that met inclusion criteria were selected for full-text abstraction, which was conducted using extraction tables. These tables included the article citation, the target study population, the study design, the study objective, the stated implementation TMF used and details regarding how the TMF was applied (Additional file 2: Table S2). When two sources were identified that reported on the same study (that is, a published conference abstract and a published manuscript), information from both sources was combined in the abstraction and analysis process. For every eligible paper, two researchers independently extracted the information. The extracted data were then compared, and discrepancies were discussed and resolved by consensus.

### Data analysis

Critical evaluation of sources of evidence was conducted using appraisal criteria outlined in the Implementation Research Development Tool (ImpRes) [25]. The ImpRes, which was developed on the basis of a literature review and expert consensus, is designed to provide guidance to IS researchers on key elements to consider when designing implementation studies. The ImpRes specifies 10 domains or criteria to consider (although not all of the domains may be equally relevant for all IS studies): (1) implementation research characteristics, (2) implementation TMFs, (3) determinants of implementation, (4) implementation strategies, (5) service and patient outcomes, (6) implementation outcomes, (7) economic evaluation, (8) stakeholders’ involvement and engagement, (9) patient and public involvement and engagement and (10) unintended consequences (Table 1).

**Table 1:** Quality appraisal criteria for evaluation of articles identified in a scoping review of the use of theories, models and frameworks in implementation science involving medicinal products Adapted from the ImpRes tool

ImpRes criteria	Appraisal definition
Domain 1: *Implementation research characteristics*	Did the authors describe where the study focus fell: that is, the degree of focus placed on reporting on the implementation process versus on evaluating the effectiveness of the intervention?
Domain 2: *Implementation theories, frameworks and models*	Because reporting regarding the use of a framework was one of the inclusion criteria for this review, this appraisal criterion was not considered.
Domain 3: *Determinants of implementation*	Did the authors report designing the study to prospectively and systematically explore factors likely to hinder or facilitate implementation efforts (for example, as outlined in CFIR)?
Domain 4: *Implementation strategies*	Did the authors report how and whether they selected specific implementation strategies to match identified barriers and facilitators?
Domain 5: *Service and patient outcomes*	Did the authors report patient and service-level outcomes (for example, admission rates) separately from implementation outcomes (conceptual distinction)?
Domain 6: *Implementation outcomes*	Did the authors report one or more implementation outcomes?
Domain 7: *Economic evaluation*	Did the authors report on the costs of different implementation strategies or provide an economic assessment of the implementation evaluation?
Domain 8: *Stakeholder involvement and engagement*	Did the authors report that other stakeholders (not patients or the public) were included as part of the implementation study design team?
Domain 9: *Patient and public involvement and engagement*	Did authors report that patients and/or the public were involved in the design of the implementation efforts?
Domain 10: *Unintended consequences*	Did authors report any unintended consequences of implementation efforts?

*CFIR* Consolidated Framework for Implementation Research, *ImpRes* Implementation Research Development Tool

Source: Implementation Research Development Tool (Hull et al. [30])

Although a comprehensive tool to appraise the conceptual and methodological quality of implementation research [Implementation Science Research Project Appraisal Criteria (ImpResPAC) tool] is under development, no quality-appraisal tool for implementation studies currently exists [39]. In light of this gap, we opted to adapt the ImpRes for this purpose. In doing so, we made two modifications: (1) we eliminated one of the quality criteria domains (that is, domain two: use of TMFs) because it duplicated our main study question, and (2) we used a dichotomous rating (that is, yes/no) to score each domain. The latter approach was used to facilitate ease of use, given that the original scoring of the ImpRes is quite lengthy and complex, requiring the rater to provide both narrative responses and to complete a series of checklists, depending on the particular domain [25]. Two researchers (MA and MYS) independently appraised each of the nine included papers using the adapted version of the ImpRes. Each cited paper was rated on the extent to which the domain content (as outlined in Table 1) had been reported (yes/no). Results were then compared, and any discrepancies between raters were resolved via discussion.

## Results

### Included studies

**Fig. 1:**
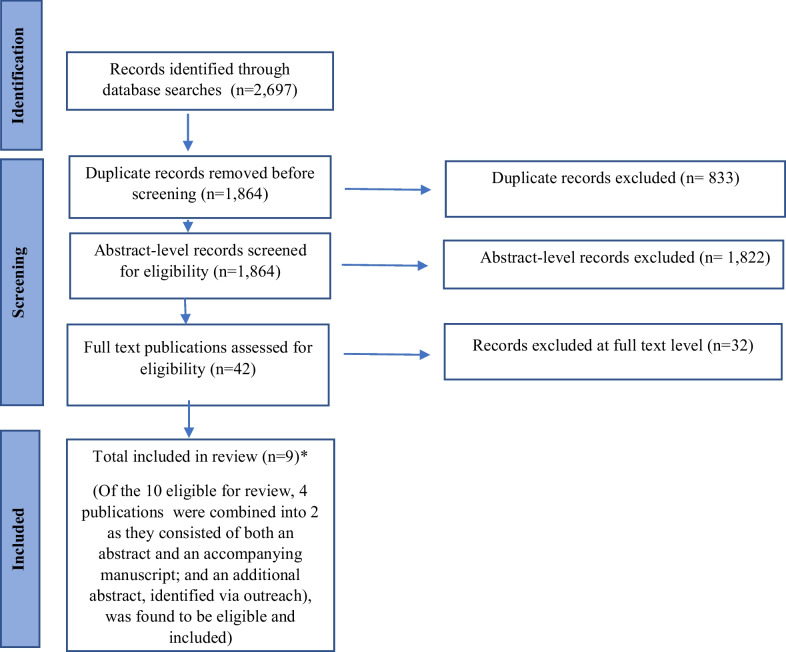
Identification and selection of publications for review and analysis

The initial search yielded 2697 citations. After removing duplicates, the total number of citations was 1864. Abstract screening identified a total of 42 articles that were eligible for full-text review. Results of the full-text review yielded 10 eligible articles; of these, 4 publications were combined into 2 because they consisted of an abstract and an accompanying manuscript. An additional abstract, identified via outreach, was also included. Figure 1 presents the results of the citation screening and review process.

### Study characteristics

Key descriptive characteristics of each study are presented in Table 2. Most eligible studies were published in 2020 or later; there was only one earlier study, dating from 2015.

**Table 2:** Characteristics of implementation science studies for medicinal products citing the use of an implementation TMF

Study citation	Study purpose	Sample size and type of participant(s)	Target disease/medical condition	Drug product	Study design	Phase in drug life cycle
Huynh et al. [49]	To evaluate FDA REMS assessment plans and FDA 2019 REMS guidance using established implementation science frameworks and identify opportunities for strengthening REMS evaluation	*n* = 23 REMS assessment plans for products that had an ETASU-type REMS *n* = 674 REMS assessment measures	No specific target condition	Products with REMS assessment plans	Retrospective content analysis using three established implementation science frameworks of REMS assessment plans was conducted for REMS programs approved by the FDA between 1 January 2014 and 31 December 2018. Framework constructs were mapped to REMS assessment categories.	Post-marketing
Jackson-Gibson et al. [27]	To evaluate implementation and program outcomes of the DREAMS intervention, including uptake and adoption of PrEP in Seme Sub-County, Kisumu, Kenya	*n* = 15 key informant interviews with Pamoja Community-Based Organization staff, healthcare providers and community leaders *n* = 40 participants in focus group discussions involving Luo-speaking young women (between 16 and 24 years of age) receiving PrEP and peer mentors	HIV	HIV PrEP	Cross-sectional study involving qualitative key informant interviews and focus groups with patients and mentors.	Post-marketing
Kalim et al. [54]	To explore the factors that influence doctors when deprescribing FRIDs in a hospital setting	*n* = 18 physicians from a large academic teaching hospital in Dublin, Ireland	Reduction of falls and fall-related injuries	Fall-risk-increasing drugs, including loop diuretics, psychotropics, opioids, antidepressants, digoxin, antiepileptics and oral hypoglycemics	Cross-sectional study involving semi-structured interviews with consultant geriatricians and hospital doctors.	Post-marketing
Meador et al. [51]	To identify/address provider-perceived barriers to optimal statin prescribing and use To use study results to inform development of interventions to help centres	*n* = 32 clinical care providers (*n* = 18 physicians; *n* = 7 physician assistants; *n* = 3 nurse practitioners; *n* = 1 clinical pharmacist; *n* = 1 nurse/RN; *n* = 2 other) from 21 health centres within 5 health centre-controlled networks across 11 US states and District of Columbia	Cardiovascular disease, specifically, statin-eligible cardiac high-risk patients	Statins	Cross-sectional survey of clinical providers.	Post-marketing
Nyeland et al. [55]	To develop a framework for evaluation of risk minimization interventions tailored to the needs of the Danish healthcare system To review its features when applied to historical data as part of a case study involving dabigatran	*n* = 21 221 Danish patients with non-valvular atrial fibrillation who had their initial prescription of dabigatran (110 or 150 mg BID doses) during the period 1 August 2011 to 30 June 2014	Non-valvular atrial fibrillation (stroke)	Dabigatran	Retrospective data review using interrupted time series analysis involving linked databases, including the Danish: (1) civil registration system, (2) national patient register and (3) national prescription drug register.	Post-marketing
Rogal et al. [53]	To assess implementation strategies that facilities used to implement case reviews To determine whether those strategies differed for facilities receiving policy notices including additional oversight from the VA national office providing implementation support To examine the association between implementation strategies, respondent characteristics, facility characteristics and case review completion rate	*n* = 89 veterans administration healthcare facilities, including rural (14%) and urban/suburban (86%)	Opioid misuse and overdose	Opioid analgesics	Randomized trial in which participating facilities were randomly assigned to receive 1 of 2 versions of a policy notice (one with oversight provisions and one without such provisions). A survey of facilities was then conducted.	Post-marketing
Sparks et al. [52]	To understand the level of awareness regarding the USP’s new patient-centred label guidelines To determine what barriers and facilitators exist to adopting these new label guidelines among key stakeholders, including pharmacists, pharmacy managers, prescribing physicians and software vendors	*n* = 5 pharmacists (*n* = 2 chain pharmacies, *n* = 3 independent pharmacies) *n* = 7 pharmacy managers (*n* = 3 at independent pharmacies, and *n* = 4 at chain pharmacies) *n* = 2 physicians *n* = 2 pharmacy software vendor representatives	All diseases and medical conditions requiring prescription drug treatment	All prescription drugs	Cross-sectional study involving semi-structured interviews with pharmacists and pharmacy managers.	Post-marketing
Toyserkani et al. [50]	To characterize REMS assessment plans using the RE-AIM framework To identify areas for advancing methods for evaluating REMS programs	*n* = 18 REMS assessment plan for REMS programs approved by the FDA between 1 January 2014 and 31 December 2018 *n* = 520 discrete measures in the REMS assessment plans	Cancers, multiple sclerosis, opioid dependence, acute pain, plaque psoriasis, opioid use disorder, generalized hypoactive sexual desire disorder, lipodystrophy, endogenous testosterone deficiency, polycystic kidney disease, phenylketonuria, polyneuropathy, paroxysmal nocturnal hemoglobinuria	Clozapine; alosetron, sodium oxybate; vigabatrin; emtricitabine/tenofovir disoproxil fumarate; metreleptin; alemtuzumab; parathyroid hormone; daclizumab; brodalumab; tisagenlecleucel; axicabtagene ciloleucel; pegvaliase; ravulizumab; testosterone undecanoate; fentanyl transdermal systems; flibanserin; tolvaptan; inotersen; sufentanil	A content analysis of REMS assessment plans. Blinded reviewers categorized each REMS assessment measure to a RE-AIM framework dimension, adjudicated their application and refined the adapted dimensions’ definitions. Dimensions were mapped to REMS assessment guidance categories.	Post-marketing
Schafer et al. [56]	To explore the real-world experiences of specialists who implemented a novel digital inhaler in clinical practice	Immunologists/allergist (*n* = 16) Pulmonologist (*n* = 1). Participants: US specialists who had prescribed a novel digital inhaler to ≥ 1 patient with asthma or chronic obstructive pulmonary disease	Patient with respiratory problems on inhaler treatment	Albuterol sulfate	Cross-sectional web-survey and qualitative interviews.	Post-marketing

*DREAMS* Determined, Resilient, AIDS-free, Mentored and Safe women; *ETASU* Elements to Assure Safe Use; *FDA* Food and Drug Administration; *FRID* fall-risk-increasing drug; *HIV* human immunodeficiency virus; *PrEP* pre-exposure prophylaxis; *RE-AIM* Reach, Effectiveness, Adoption, Implementation, and Maintenance; *REMS* Risk Evaluation and Mitigation Strategy; *TMF* theory, model, or framework; *USP* United States Pharmacopeia; *VA* Veterans Affairs

#### Study purpose

Studies varied in terms of purpose. Two studies aimed to compare the content of plans for programs to minimize drug-safety risks and the corresponding regulatory guidance pertaining to the implementation and evaluation of such programs [40, 41]. Three other studies evaluated the uptake of a drug or drug-related innovation and/or barriers and facilitators to its adoption (that is, HIV prophylaxis treatment, new patient-centred drug labelling guidelines, statin prescribing and use) [21, 42, 43]. Another study assessed implementation strategies to promote uptake and adoption of a new case-review policy for patients undergoing treatment for opioid misuse or overdose [44], whereas another study explored factors that influence doctors to deprescribe fall-risk-increasing drugs [45]. Finally, in one study, the researchers develop a framework for evaluations of programs to minimize drug-safety risks and to review its features when applied to historical data in a case study involving the drug dabigatran [46].

#### Sample size and type of participants

Study sample sizes ranged from a low of 18 (physicians) [45] to a high of 21 211 (patients) [46]. Approximately half of the studies (n = 5) had sample sizes under 50 [21, 42, 43, 45, 47]. Samples were highly diverse in type. They consisted of patients, healthcare professionals (pharmacists, prescribers, nurses and nurse practitioners and physician assistants), healthcare administrators (pharmacy managers, hospital or outpatient administrators), pharmacy software representatives, community representatives (for example, appointed leaders and youth mentors) and healthcare facilities. Two studies focused solely on evaluation of risk-minimization program measures and plans [40, 41].

#### Target disease or medical condition

Six of the studies focused on a single disease or condition (that is, HIV, opioid misuse or overdose, stroke prevention, cardiovascular disease, falls and fall-related injuries, and asthma) [21, 42, 44–47]. Three other studies involved all diseases and medical conditions that required treatment by a prescription drug, including those drugs that had Food and Drug Administration (FDA)-mandated drug safety program commitments [40, 41, 43].

#### Drug product, study design and phase in life cycle

All studies were conducted in the post-marketing phase of the drug-development cycle. Drug products featured in the studies ranged from individual therapies for the prevention or treatment of a single disease state (for example, HIV prophylaxis treatment) to products in a specific drug class (for example, statins, opioids, other fall-risk-increasing drugs such as loop diuretics, psychotropics, antidepressants, dixogin, antiepileptics and oral hypoglycaemics). Two studies pertained to all prescription drug products [40, 41], and one focused on a drug–device combination [47].

Study designs varied widely. Only one study featured a randomized clinical trial design [44]. Five studies featured cross-sectional designs involving qualitative interviews or surveys [21, 42, 43, 45, 47]. One study was a retrospective review using interrupted time-series analysis involving linked databases [46]. Two studies involved a comprehensive review and content analysis of regulatory documents [40, 41].

### Use of implementation TMFs

#### Types of implementation TMFs used, how and in which phase of research

The Consolidated Framework for Implementation Research (CFIR), a determinant framework, [34] was the most frequently used TMF (three of nine studies) [21, 40, 47] (see Table 3). Another determinant framework, the Theoretical Domains Framework (TDF), was used by Kalim et al. [45]. Rogal et al. [44] applied the Organizational Quality Index, a framework that focuses on determinants of implementation at the organizational level. Sparks et al. [43] used the classic Diffusion of Innovation Theory (DIT) to identify attributes of the intervention (a new, patient-centred drug labelling standard) that were potentially affecting its adoption.

**Table 3:** Use of implementation TMFs in implementation science studies in medicinal products

Study citation	Implementation TMF used and in which phase of research*	How TMF was used	Was a rationale for selection of TMF provided?	TMF use: full or partial	TMF domains and/or constructs used	Description of TMF domains and/or constructs used	Modifications to TMF(s)
Huynh et al. [49]	CFIR, PRECEDE-PROCEED, RE-AIM – post-implementation	QUAL	Yes	Full	All domains of RE-AIM: reach, effectiveness, adoption (both levels), implementation and maintenance (both levels). All domains of PRECEDE: social assessment; epidemiological assessment; educational/ecological assessment administrative and policy assessment and intervention alignment. All domains of PROCEED: implementation, process, impact and outcome. All domains of CFIR: innovation characteristics, outer context, inner context, characteristics of individuals and process.	Each domain within each framework was mapped to the REMS assessment FDA’s 2019 REMS guidance to determine the extent to which the domains were reflected in the REMS guidance.	None reported.
Jackson-Gibson et al. [27]	CFIR – implementation	QUAL	Yes	Full	All domains of the CFIR: innovation characteristics, outer context, inner context, characteristics of individuals and process.	The CFIR domains were used as the organizational structure for conceptualizing the study, data collection and analysis.	None reported.
Kalim et al. [54]	TDF – pre-implementation	INSTRU, QUAL, other	Yes	Partial	Six TDF domains: knowledge, skills, environmental context and resources, professional role, social influences and emotions. Domains were chosen through discussion within the research team.	To identify factors that might influence a behaviour of interest (that is, deprescribing). In this study, the interview guide consisted of 19 questions associated with the six TDF domains.	None reported.
Meador et al. [51]	PRISM – pre- and post-implementation	ID, INSTRU, QUAL and QUANT	Yes	Full	All PRISM domains: (1) intervention, (2) implementation and sustainability infrastructure, (3) recipients and (4) external environment.	PRISM constructs used to guide the intervention design were: (1) intervention, (2) implementation and sustainability infrastructure, (3) recipients and (4) external environment. The main PRISM construct that was used to guide the questionnaire: the intervention construct (both the organizational perspective and patient perspective).	None reported.
Nyeland et al. [55]	Study-specific framework: Framework for Evaluation of Effectiveness of Risk Minimization – post-implementation	IMPL, EVAL, other	Yes	Full	All four main domains: (1) data, (2) knowledge, (3) behaviour and (4) outcomes.	To guide identification of data sources (data domain), and process and outcome indicators to evaluate (that is, knowledge, behaviour and outcomes domains)	None reported.
Rogal et al. [53]	OQI – post-implementation	INSTRU	Yes	Full	All domains of OQI: (1) facility structure variables and (2) staffing/culture (for example, psychological safety).	To identify key facility characteristics to measure.	None reported.
Sparks et al. [52]	Diffusion of innovation – post-implementation	INSTRU	Yes	Partial	Only the characteristics of the innovation domain. Constructs measured within that domain included: trialability, observability, relative advantage, complexity and compatibility.	To phrase specific questions in the interview guide.	None reported.
Toyserkani et al. [50]	RE-AIM – pre-implementation	QUAL	Yes	Full	Reach, effectiveness and adoption (both levels), implementation and maintenance (both levels).	The RE-AIM domains were each compared with the content of each of the REMS assessment plans to identify areas for advancing methods for evaluating REMS programs.	Adaptation of the RE-AIM domains: using the established RE-AIM framework (20, 21); the authors created definitions applicable to REMS assessments by adapting from those defined by the framework.
Schafer et al. [56]	CFIR – post-implementation	INSTRU	None reported	Not reported	Insufficient information provided to determine.	To identify barriers and facilitators to implementation of a new digital inhaler for treatment of asthma or chronic obstructive pulmonary disease.	Insufficient information provided to determine.

*Phases of research: pre-implementation, implementation and post-implementation

*CFIR* Consolidated Framework for Implementation Research, *EVAL* evaluation of intervention, *FDA* Food and Drug Administration, *ID* intervention development; *IMPL* implementation development; *INSTRU* instrument development; *OQI* organizational quality improvement; *PRECEDE* Predisposing, Reinforcing, and Enabling Constructs in Educational/Environmental Diagnosis and Evaluation; *PROCEED* Policy, Regulatory, and Organizational Constructs in Educational and Environmental Development; *PRISM* Practical Implementation Sustainability Model; *QUAL* qualitative data analysis; *QUANT* quantitative data analysism; *RE-AIM* Reach, Effectiveness, Adoption, Implementation and Maintenance Framework; *REMS* Risk Evaluation and Mitigation Strategies; *TDF* Theoretical Domains Framework; *TMF* theory, model or framework

An evaluation framework called the Reach Effectiveness Adoption Implementation and Maintenance (RE-AIM), and its extension, the Practical, Robust Implementation and Sustainability Model (PRISM), were used in three studies [40–42]. Yet another evaluation framework, the Predisposing, Reinforcing, and Enabling Constructs in Educational Diagnosis and Evaluation – Policy, Regulatory, and Organizational Constructs in Educational and Environmental Development (PRECEDE-PROCEED), was used by Huynh et al. [40] in conjunction with CFIR. Finally, Nyeland et al. [46] developed a new evaluation framework, the Framework for Evaluation of Effectiveness of Risk Minimization.

Except for one study [40], all of the studies used only one TMF. The majority of TMFs used were multilevel in that they assessed different ecological levels, including those of the individual, the organizational setting and/or the larger community (for example, CFIR, RE-AIM, PRISM, PRECEDE-PROCEED, Diffusion of Innovation and Framework for Evaluation of Risk Minimization).

Studies varied in use across implementation phases and specific application. Six studies reported using the IS TMF after implementation of the intervention [40, 41, 43, 44, 46, 47]. Three of the post-implementation studies [43, 44, 46] used the TMFs to guide the planning of data collection tools and analysis. Two studies used TMFs to map constructs included in regulatory guidance on evaluating Risk Evaluation and Mitigation Strategy (REMS) programs, and in REMS program assessment plans [40, 41]. One study used the TMF to identify barriers and facilitators to implementation of an intervention (for example, a digital asthma inhaler) [47].

One study reported using TMFs in the pre-implementation period to identify factors that might influence a behaviour of interest (that is, deprescribing) [54]. Kalim et al. [45] used the TMF to guide the development of the interview guide, to conduct qualitative analysis of the interview data, to identify barriers in deprescribing practices and to identify potential strategies to address those barriers. Only one study reported on the use of a TMF during the implementation phase, where it was used as the organizational structure for the study [21]. Finally, Meador et al. [42] reported using the TMF in both the pre- and post-implementation phases. They used PRISM, an extension of the RE-AIM framework, to develop a questionnaire to identify and address prescriber barriers to optimize use of statin therapy.

In summary, TMFs were most frequently used to guide qualitative data coding and analysis or for qualitative instrument development. TMFs were used less frequently for the purposes of quantitative data analysis, for intervention development, for implementation planning, for intervention evaluation planning, or for “other” reasons (that is, to plan for implementation of a risk-minimization evaluation study or to identify strategies to address barriers; Table 3).

#### Rationale for TMF use

With the exception of Schafer et al. [47], all of the studies cited a specific rationale for selecting the TMF(s) that they used. These rationales were directly related either to the specific research goal, such as to evaluate a program [40–43, 46], or to address specific analytic needs, such as to analyse qualitative data pertaining to barriers to implementation [21, 44, 45, 47].

#### TMF use: full or partial

Seven of the studies used all domains or constructs of the TMF (that is, full use) [21, 40–42, 44–46]. In contrast, Sparks et al. [43] used only a single domain (the “attributes of the innovation” domain) of the Diffusion of Innovation Theory. Schafer et al. [47] did not provide sufficient information to determine the degree to which the CFIR was used.

#### TMF domains and constructs used and how applied

Studies assessed an array of domains and constructs within the TMFs. Of these, the most commonly assessed was the implementation domain included in RE-AIM, PROCEED and PRISM (Table 3). For example, Meador et al. [42] used PRISM’s implementation and sustainability infrastructure domain to develop an interview guide for prescribers to understand implementation factors affecting statin therapy uptake among high-risk patients in designated community health centres.

The innovation/intervention characteristics domain, as measured by CFIR, Diffusion of Innovation Theory, PRISM, and the PRECEDE’s intervention alignment domain, was also frequently used, typically as part of instrument development and/or qualitative data analysis. For example, Sparks et al. [43] used all six constructs from the innovation characteristics domain in the Diffusion of Innovation Theory in developing the study interview instrument.

Another domain used was CFIR’s “characteristics of individuals” implementing the innovation, a domain similar to the knowledge and behaviour domains in the Framework for Evaluating the Effectiveness of Risk Minimization [46], and by six corresponding constructs in the TDF. Kalim et al. [45] used the TDF to develop an interview guide to understand factors influencing prescribers’ deprescribing behaviours and coding of the data.

Context was also frequently assessed via one or more domains [48]. CFIR’s “process,” “inner context” and “outer setting” domains were used to understand a range of factors at different ecological levels affecting uptake of HIV PrEP and a digital asthma inhaler, respectively [21, 47]. Huyn et al. [40] used these three CFIR domains to conduct a qualitative assessment of the inclusion of contextual factors in regulatory guidance on risk minimization program evaluation. Rogal et al. [44] used the Organizational Quality Improvement framework, which focuses exclusively on organizational factors, to identify and code key facility-level covariates in a randomized trial assessing use of implementation strategies to support uptake of a new Veterans Affairs case-review policy. The “external environment” construct in PRISM, which measures a range of external environmental factors that influence intervention adoption by healthcare professionals and uptake by patients, was also used to develop a study questionnaire on prescriber uptake and sustained use of statins [42].

#### Modifications to TMFs

In most of the studies (8/9), no TMF modifications were reported. Toyserkani et al. [41] reported adapting the RE-AIM dimension definitions to enable their applicability to REMS assessments.

### Study quality appraisal

As Table 4 shows, all nine studies reported where the research fell along the implementation–effectiveness spectrum (domain 1); the majority (6/9) focused on implementation. Similarly, authors in all of the studies reported designing the study to prospectively and systematically explore factors likely to hinder or facilitate implementation efforts (domain 3). Five of the studies reported using information on implementation barriers and facilitators to design implementation strategies (domain 4) [21, 42–45]. Most of the studies (6/9) distinguished between implementation outcomes and service and patient outcomes (domain 5) [21, 40–42, 45, 47]; three studies [44, 46, 47] reported one or more implementation outcomes (domain 6). None of the studies reported on the costs of different implementation strategies (domain 7). Only one study reported on the involvement of key stakeholders (domain 8) and patients (domain 9) in the implementation efforts [21]. Lastly, only two studies [40, 41] addressed the issue of unintended consequences of implementation efforts (domain 10).

**Table 4:** Study quality appraisal using the ImpRes for implementation studies for medicinal products

ImpRes domain	Huynh et al. [49]	Jackson-Gibson et al. [27]	Kalim et al. [54]	Meador et al. [51]	Nyeland et al. [55]	Rogal et al. [53]	Sparks et al. [52]	Toyserkani et al. [50]	Schafer et al. [56]
1: Implementation research	Yes, both implementation and effectiveness	Yes, implementation	Yes, implementation	Yes, implementation	Yes, both implementation and effectiveness	Yes, implementation	Yes, implementation	Yes, both implementation and effectiveness	Yes, implementation
3: Determinants of implementation	Yes, using CFIR	Yes, using CFIR	Yes, using TDF	Yes, using PRISM	Yes	Yes, using OCF	Yes, using DIT	Yes, using RE-AIM	Yes, using CFIR
4: Implementation strategies	N/A	Yes	Yes	Yes	No	Yes	Yes	N/A	No
5: Service and patient outcomes	Yes	Yes	Yes	Yes	No	No	No	Yes	Yes
6: Implementation outcomes	Yes	Yes	Yes	Yes	No	No	Yes	Yes	No
7: Economic evaluation	No	No	No	No	No	No	No	No	No
8: Stakeholder involvement and engagement	No	Yes	No	No	No	No	No	No	No
9: Patient and public involvement and engagement	Yes	Yes	Yes	No	No	No	No	No	No
10: Unintended consequences	Yes	No	No	No	No	No	No	Yes	No

Each cited article/abstract was rated on the extent to which the domain content had been reported: Yes, reported; No, not reported. Implementation or N/A = Not applicable as it was beyond the stated study scope

ImpRes Domain 2 (TMF used) is not included because it duplicates the main goal of the research study

*CFIR* Consolidated Framework for Implementation Research; *DIT* Diffusion of Innovation Theory; *PRECEDE* Predisposing, Reinforcing, and Enabling Constructs in Educational Diagnosis and Evaluation; *ImpRes* Implementation Research Development; *OCF* Organizational Culture Framework; *PRISM* Practical Implementation Sustainability Model; *RE-AIM* Reach, Effectiveness, Adoption, Implementation and Maintenance Framework; *TDF* Theoretical Domains Framework; *TMF* theory, model, or framework

## Discussion

To the best of our knowledge, this is the first scoping review ever conducted on the use of TMFs in IS studies involving medicinal products. As awareness of the value of IS research continues to grow among both pharmaceutical company sponsors and regulators [9, 10, 15, 49], we anticipate that there will be a substantial increase in the number of such studies conducted over the next decade or so. It will be important to ensure that future studies are of high methodological quality so that results can be used to improve the efficiency with which new medicinal innovations move from the highly controlled testing environments of development to the diverse, uncontrolled conditions characteristic of real-world clinical care settings in the post-approval period. High-quality IS research is also needed to help build the evidence base regarding what implementation strategies work to support uptake and sustained use of new medicinal innovations and under what circumstances.

This paper represents an initial effort to characterize the status of medicinal product IS research at this particular inflection point. Overall, we found that the number of implementation studies involving medicinal products that reported using a TMF was extremely limited (*n* = 9). In the nine studies that did so, most used either CFIR, RE-AIM or RE-AIM’s extension, PRISM. These three are among the most widely utilized and cited implementation TMFs in the literature and hence, most likely to be familiar to IS researchers [29]. Notably, these three frameworks, along with the PROCEED-PRECEDE, measure multiple ecological levels. Thus, their application in this instance is especially appropriate given that medicinal products must be implemented into the highly complex environment of healthcare delivery, a system that features multiple types of implementation determinants operating at multiple levels. Additionally, CFIR (www.cfir.org) RE-AIM (www.re-aim.org) and PRISM are among the most well-operationalized implementation frameworks (as opposed to theories or models) available, and they have well-defined constructs, terminology, measures and guidance on use.

The foregoing factors, individually or collectively, may explain why these particular TMFs were chosen for use [35]. Given the many available implementation TMFs, selecting ones that are most appropriate for a given research project has been recognized as challenging [50]. Limited understanding of how to select a TMF can lead to its superficial application (for example, mention of the TMF in the introduction of a paper but no information provided in the methods or results sections as to how or to what extent it was applied), inappropriate use (for example, constructs not operationalized correctly) or underuse (that is, TMF use limited to just one aspect of the research as opposed to comprehensively applied throughout) [50]. Birken et al. [35] conducted a survey of 223 IS researchers to assess which TMFs they were using and how they went about selecting and applying them within their research. Both CFIR and RE-AIM were among the most cited TMFs, although more than 100 different TMFs were mentioned in all. In addition, results showed that the selection of an implementation TMF was not systematic and was as much a function of convenience or prior familiarity with the TMF as any other reason [35]. In a subsequent scoping review, Strifler et al. [32] found that IS researchers commonly failed to provide a rationale or adequate justification for their use of a particular implementation TMF.

In contrast, we found that the majority of studies in our review did provide a rationale for the TMF(s) that were used. However, their application of TMFs was typically quite limited; in the majority of instances, TMFs were used for a single purpose: either to design data collection instruments or for data analysis (either qualitative or quantitative). While most of the studies reported which TMF domains were used, few reported which specific constructs within those domains were assessed, and none reported rationales for using either the specific domains and/or constructs, as Damschroder et al. have recommended [24]. Moreover, none of the studies used TMF domains or constructs to examine associations with implementation effectiveness or other relevant outcomes, or as covariates in analytic models. For example, Kilbourne and colleagues reported using quantitative measures of CFIR constructs as control variables in predictive models to assess changes in implementation outcomes in a study of a new care engagement approach for mentally ill patients [51].

In practice, several TMFs may be amalgamated within a single research project to meet a variety of needs [52]. Only one study, Rogal et al. [44] used two TMFs, each for a different purpose (that is, instrument development and data analysis), whereas Huynh et al. [40] used three different TMFs for the purpose of mapping REMS evaluation plans. This “bricolage” approach of employing complementary TMFs within a single study can increase methodological rigour and provide richer insights into study findings, although it can also introduce greater complexity in terms of data collection, analysis and triangulation [52, 53].

Despite the state of IS practice, numerous resources regarding how to choose an implementation TMF are available. Chambers [54] has enumerated seven factors to consider when selecting an implementation model or framework, including the study aims and scope, the purpose for which the model or framework will be used, the level of socioecological change that the research is seeking to explain, the characteristics of context that are relevant to the study aims, the study time frame and whether the model or framework has available measures. Birken et al. [55] have also developed a guide for TMF selection called the Theory Comparison and Selection Tool (T-CaST), which guides the researcher through a systematic consideration of 16 different criteria when selecting a specific TMF for use for a given project.

Potentially, given that implementation research involving medicinal products is a relatively new area of study, some investigators may actually be using implementation TMFs but are neglecting to report this in publications. Such an oversight may indicate lack of awareness of IS quality reporting guidelines, which specify that the underpinning theory, framework or model should be described as part of the scientific background and study rationale [56]. To advance the science in this area, there is need for more standardized reporting of IS research, including greater transparency regarding how and why a TMF was selected. Standardized reporting can facilitate synthesis of the relevant evidence, which can, in turn, improve the speed with which effective medicinal products and related interventions achieve uptake, adoption and sustained use within the intended healthcare delivery settings [56]. Authors and journal editors alike have a role to play in ensuring that IS study results are reported adequately in the published literature – authors by adhering to such guidelines and editors by stipulating their use in the journal submission process.

Another key finding was that all the studies were conducted in the post-authorization context. In one sense, this is not surprising given that the post-marketing period is the phase in which implementation challenges related to uptake, adoption and sustainment are most publicly apparent. On the other hand, IS research is valuable in the pre-approval period as well [16, 57, 58]. In particular, it can reduce the time from drug discovery to use in clinical care, can inform product sponsors about potential barriers to uptake and adoption of their new product, and also allow for the identification of strategies to address them [16]. IS research can be also conducted during the pre-approval period to test different implementation strategies to support real-world clinical use of a product (for example, to identify and select appropriate patients or to monitor for specific side-effects). Additionally, IS research can be useful in early development (that is, pre-implementation of the medicinal product innovation) to identify potential barriers to product manufacturing and distribution [16].

In our review, the fact that none of the studies had conducted IS research prior to market launch of the medicinal product represented a lost opportunity to identify potential barriers in advance [59], and to develop or adapt their implementation strategies before implementation began [21, 42, 45, 47]. A particularly compelling example of a lost opportunity in this regard concerned the distribution of the Pfizer/BioNTech coronavirus disease 2019 (COVID-19) vaccine, which had stringent, exceptionally cold (−70 °C) storage requirements. In the absence of information regarding viable strategies for achieving rapid vaccine distribution, distribution of the vaccine proved to be complicated and experienced delays [58]. IS research can also be used in Phase 1 and 2 to explore how supportive tools or adjunctive therapies can be implemented within the upcoming Phase 3 pivotal clinical trials themselves.

In addition to reporting the use of a TMF, IS research in this area could also be improved by involving patients and other stakeholders in the conduct of implementation research, including in the selection of implementation strategies. This shortcoming is not unique to IS research involving medicinal products. As Wensing and Grol have noted, effective stakeholder engagement is a challenge currently in the field of IS generally [60]. Knowledge as to which methods are most effective for engaging patient and healthcare professionals in intervention design, implementation and evaluation is lacking, and the impact of different approaches to stakeholder engagement on outcomes has not been well studied [61].

One potential limitation of our review is that, given the relative novelty of IS in the medicinal product context, much of this research may not yet be published. Alternatively, relevant publications may have been missed because the search terms we used were for drugs and medicines broadly and not for specific medicinal products. Of the publications identified, most were excluded because, per our search strategy, they did not cite a TMF in either the title or abstract. Although we may, as a result, have missed some studies that did indeed use TMFs, our search approach was an appropriate one for the purposes of a scoping review. In addition, we sought to conduct a quality appraisal of the nine eligible studies using an adapted version of the ImpRes tool. Although the ImpRes is not as a quality-appraisal tool per se, our decision to use it in this way is justified given that it was designed to guide high-quality implementation research and no other quality appraisal options are extant.

Our scoping review highlights several areas for future research. One is the need for more IS research conducted during the preapproval phase of drug development. Initiating IS research during Phases 2 and 3 could help identify potential barriers and facilitating factors that could affect uptake, as well as what strategies might be needed to address them before the product (or related innovation) actually reaches the market. Secondly, based on the lack of studies in our review which reported cost estimates, future research should focus on collecting and reporting on the cost–effectiveness of using different implementation strategies. Given the likely underreporting of TMFs in IS research for medicinal products, a third topic of research would be to interview IS researchers working in this area currently to determine what TMFs are being used, how they were selected, and how they are being applied, similar to earlier research by Birken et al. [35] in the field of IS in general.

## Conclusion

We found that few IS studies involving medicinal products reported using TMFs. Those that did encompassed a wide variety of therapeutic indications and medicinal products; all were in the post-marketing phase and involved limited application of the TMF. Researchers should consider conducting IS in earlier phases of drug development and integrate the TMF throughout the research process. More consistent and in-depth use of TMFs may help advance implementation science research in this area.

### Supplementary Information

**Additional file 1: Table S1.** Search terms used in scoping review of use of theories, models and frameworks in implementation science studies involving medicinal products.

**Additional file 2: Table S2.** Data extraction fields for scoping review of the use of theories, models and frameworks in implementation science studies involving medicinal products.

## Data Availability

Available upon request to the corresponding author.

## Abbreviations

IS: Implementation science
HIV: Human immunodeficiency virus
TMFs: Theories, models, and frameworks
CFIR: Consolidated Framework for Implementation Research
TDF: Theoretical Domains Framework
PRISMA: Preferred Reporting Items for Systematic Reviews and Meta-Analyses
ImpResPAC: Implementation Science Research Project Appraisal Criteria
FDA: Food and Drug Administration
RE-AIM: Reach, Effectiveness, Adoption, Implementation, and Maintenance
PRISM: Practical, Robust Implementation and Sustainability Model
PRECEDE-PROCEED: Predisposing, Reinforcing, and Enabling Constructs in Educational Diagnosis and Evaluation–Policy, Regulatory, and Organizational Constructs in Educational and Environmental Development
REMS: Risk Evaluation and Mitigation Strategy
T-CaST: Theory Comparison and Selection Tool

## Declarations

### Declarations

MYS, BG and MA are full time employees of Evidera, Inc., a business unit of Pharmaceutical Product Development (PPD), a healthcare research consultancy. They are also shareholders in Thermo Fisher Scientific, which owns PPD. MYS is an adjunct faculty member in the Department of Regulatory and Quality Sciences, School of Pharmacy, University of Southern California, Los Angeles, CA.
