# Supplementary material for: The use of implementation science theories, models, and frameworks in implementation research for medicinal products: A scoping review
Source: Health Res Policy Syst. 2024 Jan 29;22:17. doi: 10.1186/s12961-024-01102-0 (PMC10823700; doi:10.1186/s12961-024-01102-0)
Supplement: Supplementary file 1 — Additional file 1: Table S1. Search terms used in scoping review of use of theories, models and frameworks in implementation science studies involving medicinal products. [file 12961_2024_1102_MOESM1_ESM.docx]

Additional file 1: Table S1. Search terms used in scoping review of implementation research involving medicinal products.

Embase (Ovid): 1974 to 2022 December 12. Date searched: 13 December 2022

| Search Number | Search Terms | Results |
| --- | --- | --- |
| 1 | *implementation science/ | 1429 |
| 2 | *diffusion of innovation/ | 40 |
| 3 | *translational research/ | 7637 |
| 4 | (implementation adj3 (science or research or effect or effects or effectiveness or framework$ or theory or theories or model or models or modelling or evaluation$ or process$ or construct$ or domain$ or facilitate$ or scaling up or scale up)).ti,ab. | 46574 |
| 5 | (diffusion adj3 innovation).ti,ab. | 788 |
| 6 | (CFIR or RE-AIM or Pragmatic Robust Implementation).ti,ab. | 1936 |
| 7 | Precede-Proceed.ti,ab. | 294 |
| 8 | ((knowledge or biomedical or science) adj3 translation$).ti,ab. | 9461 |
| 9 | or/1-8 | 63930 |
| 10 | exp *drug development/ | 41050 |
| 11 | ((drug or drugs or pharmaceutical$ or medication$ or medicine or medicinal) adj3 (development$ or discover$ or continuum or lifecyle or life cycle or target prediction$ or innovation $ or pipeline or accelerat$)).ti,ab. | 164875 |
| 12 | ((drug or drugs or pharmaceutical$ or medication$ or medicine or medicinal) adj3 (safety or risk or risks)).ti,ab. | 64790 |
| 13 | ((risk or risks or mitigation) adj3 (strateg$ or program$ or initiative$ or evaluat$ or minimi?ation)).ti,ab. | 109208 |
| 14 | or/10-13 | 361134 |
| 15 | 9 and 14 | 1801 |
| 16 | (exp animal/ or nonhuman/) not exp human/ | 6952493 |
| 17 | (editorial or letter or note).pt. or in vitro study/ or (commentary or editorial or comment or letter or mice or rat or mouse or animal or murine).ti. | 5594451 |
| 18 | 15 not (16 or 17) | 1597 |
| **19** | **limit 18 to (english language and yr="2000 -Current")** | **1494** |

MEDLINE-ALL (Ovid): 1946 to December 12, 2022. Date searched: 13 December 2022

| Search Number | Search Terms | Results |
| --- | --- | --- |
| 1 | exp *"diffusion of innovation"/ | 10222 |
| 2 | *Translational Science, Biomedical/ | 86 |
| 3 | (implementation adj3 (science or research or effect or effects or effectiveness or framework$ or theory or theories or model or models or modelling or evaluation$ or process$ or construct$ or domain$ or facilitate$ or scaling up or scale up)).ti,ab. | 38046 |
| 4 | (diffusion adj3 innovation).ti,ab. | 731 |
| 5 | (CFIR or RE-AIM or Pragmatic Robust Implementation).ti,ab. | 1761 |
| 6 | Precede-Proceed.ti,ab. | 267 |
| 7 | ((knowledge or biomedical or science) adj3 translation$).ti,ab. | 7248 |
| 8 | or/1-7 | 54857 |
| 9 | exp *Drug Development/ | 48318 |
| 10 | ((drug or drugs or pharmaceutical$ or medication$ or medicine or medicinal) adj3 (development$ or discover$ or continuum or lifecyle or life cycle or target prediction$ or innovation $ or pipeline or accelerat$)).ti,ab. | 125372 |
| 11 | ((drug or drugs or pharmaceutical$ or medication$ or medicine or medicinal) adj3 (safety or risk or risks)).ti,ab. | 41560 |
| 12 | ((risk or risks or mitigation) adj3 (strateg$ or program$ or initiative$ or evaluat$ or minimi?ation)).ti,ab. | 76246 |
| 13 | or/9-12 | 275625 |
| 14 | 8 and 13 | 1130 |
| 15 | exp animals/ not humans/ | 5073054 |
| 16 | (comment or editorial or introductory journal article or letter or news or newspaper article or overall).pt. or In Vitro Techniques/ or (commentary or editorial or comment or letter or mice or rat or mouse or animal or murine).ti. | 3963243 |
| 17 | 14 not (15 or 16) | 1065 |
| 18 | limit 17 to english language | 1025 |
| **19** | **limit 18 to yr="2000 -Current"** | **987** |

**CENTRAL (Cochrane Central Register of Controlled Trials. EBM Reviews) (Ovid): November 2022. Date searched: 13 December 2022**

| Search Number | Search Terms | Results |
| --- | --- | --- |
| 1 | exp *"Diffusion of Innovation"/ | 0 |
| 2 | *Translational Science, Biomedical/ | 0 |
| 3 | (implementation adj3 (science or research or effect or effects or effectiveness or framework$ or theory or theories or model or models or modelling or evaluation$ or process$ or construct$ or domain$ or facilitate$ or scaling up or scale up)).ti,ab,kw. | 5198 |
| 4 | (diffusion adj3 innovation).ti,ab,kw. | 59 |
| 5 | (CFIR or RE-AIM or Pragmatic Robust Implementation).ti,ab,kw. | 546 |
| 6 | Precede-Proceed.ti,ab,kw. | 67 |
| 7 | ((knowledge or biomedical or science) adj3 translation$).ti,ab,kw. | 585 |
| 8 | or/1-7 | 5953 |
| 9 | exp *Drug Development/ | 61 |
| 10 | ((drug or drugs or pharmaceutical$ or medication$ or medicine or medicinal) adj3 (development$ or discover$ or continuum or lifecyle or life cycle or target prediction$ or innovation $ or pipeline or accelerat$)).ti,ab,kw. | 2761 |
| 11 | ((drug or drugs or pharmaceutical$ or medication$ or medicine or medicinal) adj3 (safety or risk or risks)).ti,ab,kw. | 61122 |
| 12 | ((risk or risks or mitigation) adj3 (strateg$ or program$ or initiative$ or evaluat$ or minimi?ation)).ti,ab,kw. | 5628 |
| 13 | or/9-12 | 68844 |
| 14 | 8 and 13 | 181 |
| **15** | **limit 14 to yr="2000 -Current"** | **180** |

**Cochrane Database of Systematic Reviews (CDSR. EBM Reviews) (Ovid): 2005 to December 7, 2022. Date searched: 13 December 2022**

| Search Number | Search Terms | Results |
| --- | --- | --- |
| 1 | (implementation adj3 (science or research or effect or effects or effectiveness or framework$ or theory or theories or model or models or modelling or evaluation$ or process$ or construct$ or domain$ or facilitate$ or scaling up or scale up)).ti,ab,kw. | 32 |
| 2 | (diffusion adj3 innovation).ti,ab,kw. | 1 |
| 3 | (CFIR or RE-AIM or Pragmatic Robust Implementation).ti,ab,kw. | 1 |
| 4 | Precede-Proceed.ti,ab,kw. | 0 |
| 5 | ((knowledge or biomedical or science) adj3 translation$).ti,ab,kw. | 3 |
| **6** | **or/1-5** | **36** |

**Total = 2697**

**Total after removal of duplicates = 1864**
