# Supplementary material for: The use of implementation science theories, models, and frameworks in implementation research for medicinal products: A scoping review
Source: Health Res Policy Syst. 2024 Jan 29;22:17. doi: 10.1186/s12961-024-01102-0 (PMC10823700; doi:10.1186/s12961-024-01102-0)
Supplement: Supplementary file 2 — Additional file 2: Table S2. Data extraction fields for scoping review of the use of theories, models and frameworks in implementation science studies involving medicinal products. [file 12961_2024_1102_MOESM2_ESM.docx]

Additional file 2: Table S2. Data extraction fields for scoping review of the use of theories, models, and frameworks in implementation science studies involving medicinal products.

| **Field** | **Information Extracted** |
| --- | --- |
| Study Citation | Full Citation of source, year of publication abstracted separately |
| Sample Size | Number of participants in the study |
| Sample Description | Characteristics of the sample (patients, providers, distinct characteristics) |
| Target disease/ medical condition | Description of the specific indication of the study |
| Drug Product | Name of drug product |
| Study Purpose | Objective of the study as stated by authors |
| Study Design | Description of study design |
| Phase in Drug Lifecycle | Discovery and development, clinical research, drug review, post-marketing |
| Implementation TMF Used | Exact description of TMF as provided by authors |
| Phase of the research TMF used | Pre-implementation; Implementation; post-implementation |
| Rationale for Selection | Reasons for selection of TMF provided by authors |
| Level of use of TMF | Full TMF used/Partial TMF use |
| Specific application of TMF | 1) Intervention development; 2) Construct Identification; 3) Implementation development; 4) Instrument Development; 5) Qualitative Data Analysis; 6) Quantitative Data Analysis; 7) Not Reported how |
| List of constructs/domains used in study | Full list of domains used |
| How were each construct/domain used? | Description of use |
| Modifications/Adaptations Made to Framework | Yes/No |
